# Improved *Diaphorina citri* (Hemiptera: Liviidae) Adults Biocontrol in Citrus by *Hirsutella citriformis* (Hypocreales: Ophiocordycipitaceae) Gum-Enhanced Conidia Formulation

**DOI:** 10.3390/plants12061409

**Published:** 2023-03-22

**Authors:** Orquídea Pérez-González, Ricardo Gomez-Flores, Roberto Montesinos-Matías, Marco A. Mellín-Rosas, Servando H. Cantú-Bernal, Patricia Tamez-Guerra

**Affiliations:** 1 Departamento de Microbiología e Inmunología, Facultad de Ciencias Biológicas, Universidad Autónoma de Nuevo León, San Nicolás de los Garza C.P. 66451, NL, Mexico; 2 Centro Nacional de Referencia de Control Biológico, SENASICA, Tecomán C.P. 28110, Col., Mexico

**Keywords:** fungi culture, higher spore yields, solid fermentation, *Acacia* gum, gum production, conidia production

## Abstract

*Hirsutella citriformis* Speare is the only entomopathogenic fungus involved in *Diaphorina citri* Kuwayama natural epizootics. The aim of the present study was to evaluate different protein sources as supplements to stimulate *Hirsutella citriformis* growth, improve conidiation on solid culture, and evaluate its produced gum for conidia formulation against *D. citri* adults. *Hirsutella citriformis* INIFAP-Hir-2 strain was grown on agar media enriched with wheat bran, wheat germ, soy, amaranth, quinoa, and pumpkin seed, in addition to oat with wheat bran and/or amaranth. The results demonstrated that 2% wheat bran significantly (*p <* 0.05) promoted mycelium growth. However, 4% and 5% wheat bran achieved the highest conidiation (3.65 × 10^7^ conidia/mL and 3.68 × 10^7^ conidia/mL, respectively). Higher conidiation (*p <* 0.05) was observed on oat grains supplemented with wheat bran, as compared with culturing on oat grains without supplements (7.25 × 10^7^ versus 5.22 × 10^7^ conidia/g), after a 14 d instead of 21 d incubation period. After supplementing synthetic medium or oat grains with wheat bran and/or amaranth, INIFAP-Hir-2 conidiation increased, whereas production time was reduced. After using *Acacia* and *Hirsutella* gums to formulate conidia produced on wheat bran and amaranth at 4%, field trial results showed that the highest (*p <* 0.05) *D. citri* mortality was achieved by *Hirsutella* gum-formulated conidia (80.0%), followed by the *Hirsutella* gum control (57.8%). Furthermore, *Acacia* gum-formulated conidia caused 37.8%, whereas *Acacia* gum and negative controls induced 9% mortality. In conclusion, *Hirsutella citriformis* gum used to formulate its conidia improved biological control against *D. citri* adults.

## 1. Introduction

The Asian citrus psyllid or Citrus psylla *(Diaphorina citri* Kuwayama) causes the most serious disease of citrus in Mexico and worldwide [1]. Its lifecycle ranges from 15 d to 47 d, depending on weather conditions, and adults live a few months. It produces 9 to 10 generations yearly, whose eggs hatch from 2 d to 4 d and five instars are completed within 11 d to 15 d [2]. Both nymphs and adults are sucking insects, leading to leaves curling, defoliation, flowers falling, and branch death. The resulting damage is known as dieback [1]. *Diaphorina citri* Kuwayama, a vector of Ca. Liberibacter, is the causal agent of Huanglongbing (HLB), which is susceptible to *Hirsutella* infection [1,3,4,5]. HLB is considered one of the most devastating diseases of cultivated citrus species globally, causing important economic losses [6,7]. To date, more than 60 million trees have been affected [8]. In South Africa, damages amounting to 100% of crop losses were reported, whereas in Brazil and the USA, HLB has caused significant citrus production reduction [9,10]. In Mexico, HLB infection has caused a 42% and 33% decrease in orange and lemon production, respectively [11]. Recently, strategies for managing *Candidatus Liberibacter* spp. have been developed, including broad-spectrum antimicrobials, *Ca*. Liberibacter asiaticus-specific anti-microbials, thermotherapy, plant growth promoters, and/or host defense boosters [12]. In addition, diverse control measures have been implemented, including plant protection practices, and chemical and biological agents such as natural enemies and entomopathogenic fungi, which are considered significant insect biological control agents [13]. In this regard, *Hirsutella* is one of the most abundant and important fungal genera for pest insect control in the field. It includes about 90 species infecting and parasitizing a wide variety of invertebrates such as mites and insects, many of which are considered pests of economic importance [14].

*Hirsutella citriformis* Speare is a difficult-to-grow-complex fungus with limited shelf-life, which develops a low spore amount compared with other entomopathogenic fungi [15]. Although its biological control potential against *Diaphorina citri* is recognized, its application as a tool in plant protection programs has not yet been established. Moreover, to enhance conidia production, a protein source, such as yeast extract, must be supplemented to the culture medium [16]. If *Hirsutella citriformis* is expected to be used as a biopesticide, identification of low-cost protein (nitrogen) sources and natural substrates is necessary to stimulate growth and conidiation. Such an inexpensive natural protein supplements is found in different grains and seeds.

Substrates often used for entomopathogenic fungi conidia production include rice (*Oryza sativa* L.), barley (*Hordeum vulgare* L.), beans (*Phaseolus vulgaris* L.), maize (*Zea mays* L.), oat (*Avena sativa* L.), sorghum (*Sorghum bicolor* [L.] Moench), soybean (*Glycine max* [L.] Merr.), and wheat (*Triticum aestivum* L.) grains [17,18].

*Hirsutella* infection begins after conidia adhere to target insect’s cuticle, germinate, and pseudomycelium penetrate the hemocele. Once inside, mycelium develops from insect tissues, which leads to the insect’s death. After the insect dies, mycelium protrudes [19,20]. *Hirsutella citriformis* produces a mucilaginous conidium envelope (gum), which facilitates its adherence and provides protective potential to temperatures above or below the optimum, ultraviolet radiation, cold stress, or high osmotic pressure exposure [20,21]. During developing, most fungi produce secondary metabolites, some of which may act as insect toxins. Among *Hirsutella* species, the most recognized is hirsutellin A (HTA), which was isolated from *H. thompsonii* F.E. Fisher [22,23].

The aim of the present study was to evaluate different protein sources as supplements to stimulate *Hirsutella citriformis* growth and improve conidiation and gum production on solid culture media [24]. In addition, the toxins present in the gum produced by *Hirsutella* may increase this fungus virulence against target insects [20,25]. Thus, formulation of *Hirsutella citriformis* gum and conidia may increase *Diaphorina citri* adults´ biocontrol as applied. *Acacia* gum has been used to formulate fungi conidia in the past for formulation purposes [11,26]. We predicted that a formulation with *Acacia* or *Hirsutella* gum (used to formulate *Hirsutella citriformis* conidia) produced on selected grains, is effective to control *Diaphorina citri* adults on citrus, under laboratory and field conditions.

## 2. Results

### 2.1. Radial Growth and Conidiation in Agar

*Hirsutella citriformis* strain INIFAP-Hir-2 radial growth and conidiation on culture media are shown in Table 1. In the first experiment, the radial growth average of flour-cultured strains ranged from 2.9 to 3.6 cm. After testing the same strain among extracts added as supplements, radial growth averaged from 2.5 to 3.7 cm. The strain’s highest growth (*F* = 9.941; df = 24, 50; *p <* 0.001) was observed on PDAY supplemented with 4% soybean, amaranth, quinoa, wheat bran, and wheat germ flours or pumpkin seed, wheat bran, and quinoa extracts, whereas the highest radial growth was achieved using 4% wheat germ and 2% wheat bran extracts as supplements (Table 1).

However, the highest conidiation (*F* = 5.49; df = 24, 50; *p <* 0.001) was observed after culturing on 4% wheat bran flour (36.9 × 10^6^ conidia/mL), whereas the lowest production was detected on 4% soy extract supplement (12.1 × 10^6^ conidia/mL) (Table 1).

**Figure 1:**
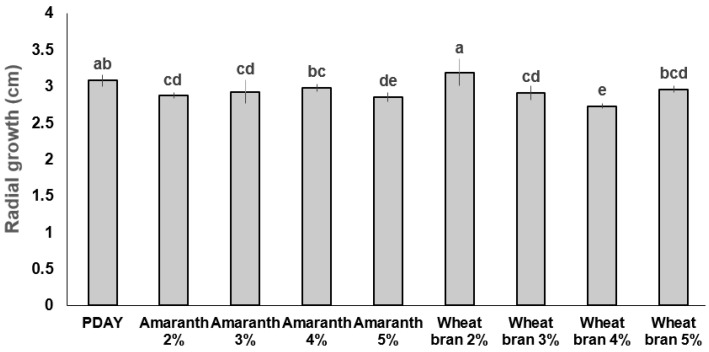
Mexican *Hirsutella citriformis* Strain INIFAP-Hir-2 radial growth. Fungi radial growth (cm) was measured after 35 d incubation at 25 °C on agar media. PDAY bar represents potato dextrose agar + 1% yeast extract culture. The other bars represent dextrose, peptone, and yeast extract agar (DPYA), supplemented with amaranth or wheat bran at 2%, 3%, 4%, and 5%. Different letters on each column indicate significant statistical differences, and bars indicate the standard error of the mean (Tukey test).

**Figure 2:**
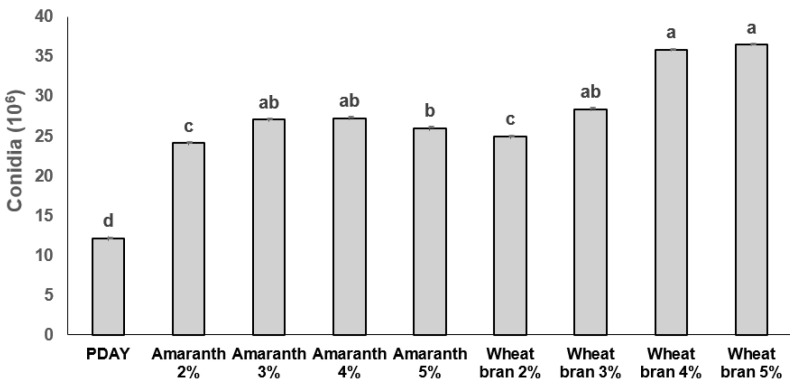
*Hirsutella citriformis* conidia production on protein supplements. Fungi conidia production was determined after culturing with protein supplements, as explained in the text. The PDAY bar represents potato dextrose agar + 1% yeast extract culture. The other bars represent dextrose, peptone, and yeast extract agar (DPYA), supplemented with amaranth or wheat bran at 2%, 3%, 4%, and 5%. Different letters indicate significant statistical differences, and bars indicate the standard error of the mean (Tukey test).

In the second experiment, the highest *Hirsutella citriformis* radial growth (F_8,36_ = 9.409; *p <* 0.001) was observed on 2% wheat bran supplement (3.19 cm) and PDAY (3.08 cm), compared with 2% amaranth (2.87 cm) and 4% wheat bran (2.73 cm) supplements (Figure 1). In addition, the highest conidia production (F_8,36_ = 44.47; *p <* 0.001) was obtained after adding 5% (36.45 × 10⁶), followed by 4% (35.8 × 10⁶), and 3% (28.4 × 10⁶) wheat bran as supplements (Figure 2).

### 2.2. Conidia Production on Oat

**Figure 3:**
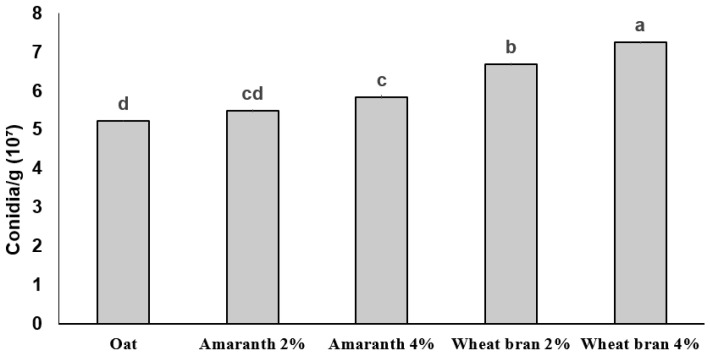
*Hirsutella citriformis* conidia production on oat grains. The Oat bar represents oat used as substrate or oat supplemented with amaranth or wheat brand at 2% and 4%. Fungi conidia production per gram of oat with or without different protein sources was evaluated at 14 d, as detailed in the text. Different letters indicate significant statistical differences, and bars indicate the standard error of the mean (Tukey test).

Conidia production on oat plus supplements reached up to 10⁷ conidia/g (Figure 3). The highest conidia production by INIFAP-Hir-2 was observed after culturing on oat with wheat bran after a 14 d incubation period, whereas the lowest one was detected on oat without supplements (5.22 × 10⁷ conidia/g). Furthermore, the highest production was obtained after culturing on oat supplemented with 4% wheat bran (7.25 × 10⁷ conidia/g) (F_4,10_ = 40.167; *p <* 0.001) (Figure 3).

### 2.3. Hirsutella Citriformis Gum Production

**Figure 4:**
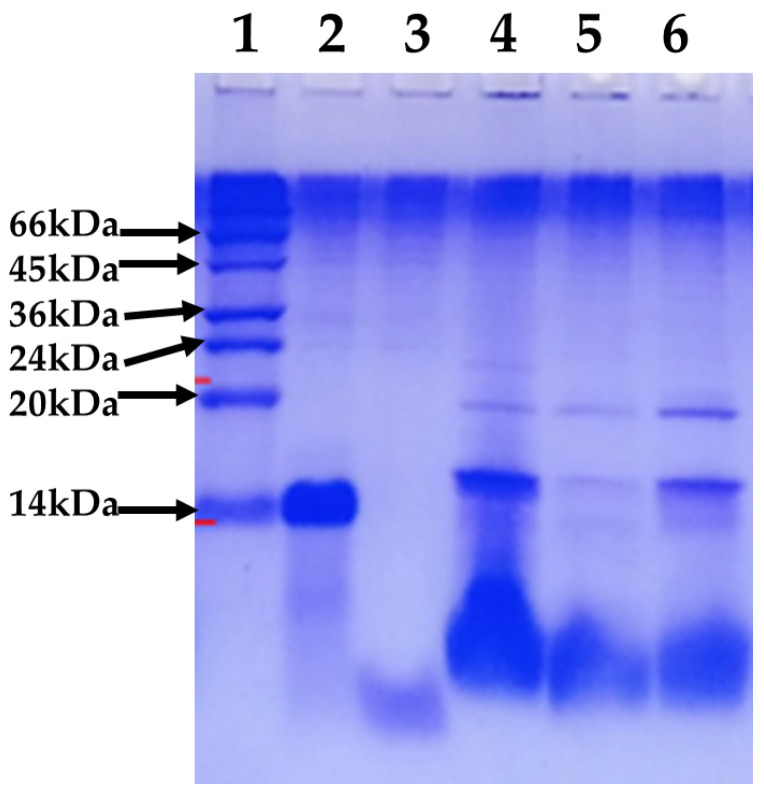
Polyacrylamide gel electrophoresis of Mexican *Hirsutella citriformis* OP-Hir-10 strain gum. Lane 1 = molecular size marker; lane 2 = supernatant; lane 3 = gum dissolved in solvent (sample was off the gel lane); lane 4 = mycelium; lane 5 = mycelium + gummous material dissolved in solvent and sonicated (five pulses/40 W/30 s); lane 6 = sonicated gummous material.

Polyacrylamide gel electrophoresis of OP-Hir-10 Mexican *Hirsutella citriformis* strain gum, showing proteins between ~14 and ~20 kDa size (Figure 4).

### 2.4. Laboratory Bioassays

**Figure 5:**
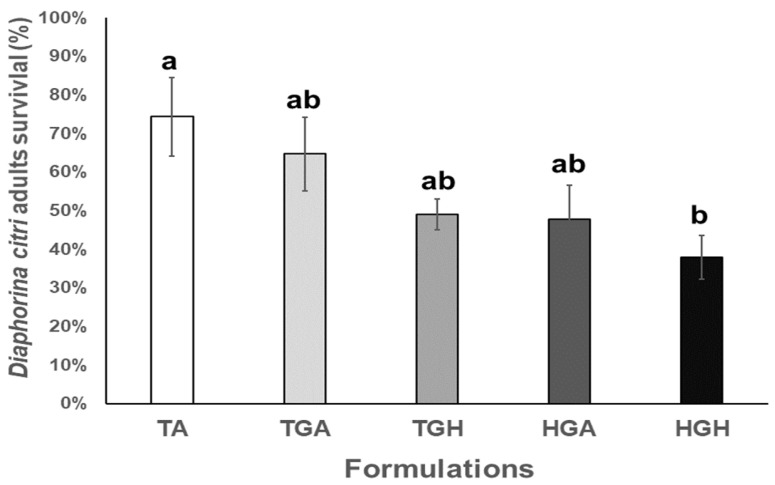
*Diaphorina citri* adults’ survival percentage average in laboratory bioassay after *Hirsutella citriformis* formulated conidia application by spraying. Absolute control = TA, *Acacia* gum control = TGA, *Hirsutella* gum control = TGH, formulation with *Acacia* gum = HGA, formulation with *Hirsutella* gum = HGH, the Hirsutella gum control (TGH) presented a mortality of 57.8%. Different letters indicate significant statistical differences, and bars indicate the standard error of the mean (Tukey test).

*Diaphorina citri* adults mortality results showed significant differences between the *formulations* and absolute control treatments in laboratory bioassays (F_4,20_ = 3.394; *p <* 0.028). The highest mortality was observed by the HGH formulation, which infected and killed 65.8% of treated insects (Figure 5). Similarly, the only treatment that showed a lower lethal time was HGH formulation (13.2 d; CL_95_ = 11.367–15.033) (Supplementary Figure S1 and Table S1).

### 2.5. Field Assays

**Figure 6:**
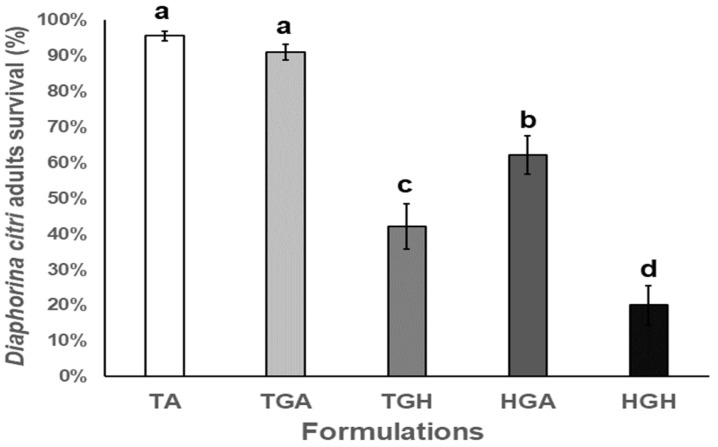
*Diaphorina citri* adults’ survival average in field after *Hirsutella citriformis* formulated conidia application by spraying. Absolute control = TA, *Acacia* gum control = TGA, *Hirsutella* gum control = TGH, formulation with *Acacia* gum = HGA, formulation with *Hirsutella* gum = HGH, the Hirsutella gum control (TGH) presented a mortality of 57.8%. Different letters indicate significant statistical differences, and bars indicate the standard error of the mean (Tukey test).

The first field trial results indicated that conidia treatments with *Acacia* gum (HGA) and *Hirsutella* gum (HGH) showed pathogenicity against *Diaphorina citri* adults in the range of 62.2% to 20.0% survival by HGA and HGH treatments, respectively (F_4,25_ = 48.748; *p <* 0.001). *Hirsutella* gum control (TGH) showed 42.2% survival. The Hirsutella gum control (TGH) presented a mortality of 57.8%. *Hirsutella citriformis* gum alone (TGH formulation) resulted in 44% insect survival. *Hirsutella citriformis* did not colonize orange plants during the evaluated period. Untreated and *Acacia* gum controls showed the lowest mortality (up to 9%) (Figure 6).

**Figure 7:**
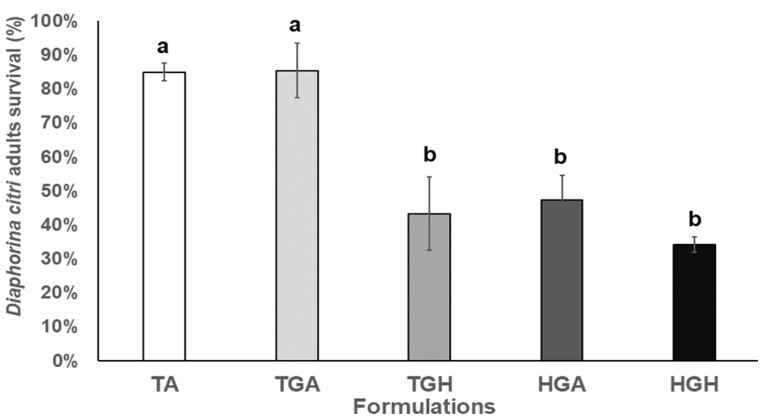
*Diaphorina citri* adults’ survival average in field after *Hirsutella citriformis* formulated conidia application by spraying. Absolute control = TA, *Acacia* gum control = TGA, *Hirsutella* gum control = TGH, formulation with *Acacia* gum = HGA, formulation with *Hirsutella* gum = HGH, the Hirsutella gum control (TGH) presented a mortality of 57.8%. Different letters indicate significant statistical differences, and bars indicate the standard error of the mean (Tukey test).

The second field trial results were similar than those collected from the first trial, where conidia treatments with *Acacia* gum (HGA) or *Hirsutella* gum (HGH) showed the highest pathogenicity against *Diaphorina citri* adults (47.2% to 34.2% survival) by HGA and HGH treatments, respectively (F_4,10_ = 11.961; *p* = 0.001). *Hirsutella* gum control (TGH) showed 43.3% survival. The Hirsutella gum control (TGH) presented a mortality of 57.8%. Absolute and *Acacia* gum controls showed the lowest mortality (up to 85.3% survival) (Figure 7). Dead insects percentage, which developed aerial mycelium after field application treatments, ranged from 6.5% to 17.3%.

## 3. Discussion

Conidia production by entomopathogenic fungi is a crucial factor to consider if they are intended for inoculative-inundative pests’ biological control. Fungi are known to grow on a wide variety of organic and inorganic nitrogen sources [18]. In this regard, there are reports on diverse nitrogen sources to stimulate entomopathogenic fungi growth and sporulation [18,27,28].

An effective and low-cost nitrogen source selection for achieving *Hirsutella citriformis* optimal growth is required for large-scale blastospores and conidia production. In this study, inexpensive high-protein content grains and seeds were selected as supplements to stimulate *Hirsutella citriformis* growth and conidiation. The highest growth and conidiation were obtained by adding natural wheat bran extract and wheat bran flour, respectively. Conidial yield did not correlate with colony growth, which agreed with previous reports [27,29].

INIFAP-Hir-2 strain reached 17.5 × 10^6^ conidia/mL on PDA supplemented with 1% yeast extract, whereas culturing on PDA supplemented with 4% natural wheat bran flour significantly increased conidia production (36.9 × 10^6^ conidia per Petri dish, adding 10 mL of water). In contrast, a production of 1.15 × 10^8^ conidia per Petri dish was reported after culturing INIFAP-Hir-2 on PDA enriched with 50% quinoa but using only 5 mL to recover conidia from the Petri dish [24].

Rice is the most used substrate for entomopathogenic fungi mass production for maintaining physical conditions and providing an effective and suitable surface for mycelial growth. After testing oat and rice to produce *H. citriformis* conidia, we obtained yields of 5.85 × 10⁷ conidia/g and 1.95 × 10⁷ conidia/g, respectively [15,29]. However, culture on oat lasted seven more days to reach the highest conidial production. We also observed higher conidia production on oat, compared with that on rice as substrate in solid culture.

In addition, we used a diphasic culture on natural substrates to stimulate fungi growth and conidia production. To achieve this, fungi were first cultured on 100 g of oat grains in 500 mL plastic containers for 14 d. Harvested conidia were then used to inoculate 500 g of oats grains and cultured in polypropylene bags. Furthermore, lower conidia production was observed after inoculating INIFAP-Hir-2 strain at higher volume and conidia concentration, and using a longer incubation time (28 d), as compared with that in our study [24]. The use of polypropylene bags may provide better conditions for accelerated fungus conidiation. They have a large surface area for increased light radiation [15]. Based on our results, these culture characteristics favor fungus growth and conidia production, and reduce incubation time for an optimum conidiation.

It has been reported that the carbon source in the culture medium is essential to stimulate fungus sporulation, whereas nitrogen content is the growth-limiting factor [28,29]. In fact, by increasing the nitrogen amount, culture time was reduced from 21 d to 14 d [15]. Similarly, a relationship between nitrogen amount and conidiation was observed in this study [18]. The highest conidia production was observed on oats supplemented with wheat bran (Figure 3), similar to that reported testing *Lecanicillium lecanii* R. Zare & W. Gams conidia production, being higher when cultured on oat grains supplemented with wheat bran [30]. After adding carbon and protein sources to sorghum, comparable results were recorded by producing *Metarhizium anisopliae* (Metchnikoff) Sorokin conidia, compared with that produced on rice [31,32]. As a supplement, wheat brat contains 15.55 g of protein and 21.72 g of carbohydrates for every 100 g, plus B3, B4, B9, and E vitamins and minerals such as iron and potassium. These nutrients may have played a role in increasing *Hirsutella citriformis* conidiation. In addition, wheat bran is considered an inexpensive supplement and its use in solid culture may result in 39% higher *Hirsutella citriformis* conidiation.

*Hirsutella citriformis*-produced gum after culturing on oat and wheat bran at 4%, and using it to formulate this fungus conidia, was observed to increase *Diaphorina citri* adults’ mortality by the OP-Hir-10 strain, as compared with previous field experiments. This result may be related to the toxicity of *Hirsutella*-produced gum to *Diaphorina citri* adults, thus showing an additive (conidia + gum) effect. Gum analysis revealed proteins with a molecular weight of ~14 and ~20 kDa, which may be related to oosporins (also reported by *Hirsutella* spp.), whose activity is associated with female’s egg reduction [33] and hirsutellin [20], respectively.

Regarding the laboratory experiments, in both trials, treatments including formulated conidia showed pathogenicity against *Diaphorina citri* adults, where the gums used in formulations demonstrated that *Hirsutella citriformis* produced gum alone killed 50% of the insects, thus indicating that this gum seems to have some detrimental effect on insect adults. The mortality on *Diaphorina citri* adults achieved by the OP-Hir-10 strain applied by spraying using *Hirsutella citriformis* gum was higher (80%) than that reported on previous assays after testing four *H. citriformis* strains, also applied by spraying (35.72% to 51.05%), whose mortality was similar to this trial by using *Acacia* gum in the formulation (37.8%) [5].

Overall, *D. citri* adults’ mortality in this trial was higher compared with the mortality rates reported in field trials using inoculation by contact [11]. In the present study, limited aerial mycelium from the infected insects were collected in the field. This may be related to the experiment environmental conditions, since the relative humidity recorded during the bioassay was sufficient for *Hirsutella citriformis* to infect and kill *Diaphorina citri* adults, but not for the fungus to develop aerial mycelium after the insects’ death. Similarly, low mycosis in the field under similar relative humidity conditions was reported during other field trials [24].

## 4. Materials and Methods

### 4.1. Fungi Radial Growth and Sporulation on Agar Media

Grains and seeds used as proteins supplement were selected based on their protein content (Table 2). Tested culture media included potato dextrose agar (PDA) supplemented with 0.5% yeast extract (PDAY) or dextrose, peptone, and yeast extract agar (DPYA) medium containing 20 g/L dextrose, 10 g/L peptone, 10 g/L yeast extract, one supplement (wheat bran, wheat germ, soybean, amaranth (*Amaranthus* spp.), or quinoa (*Chenopodium quinoa* Willd.), and pumpkin seeds (*Cucurbita moschata* Duchesne) at 20 g/L and 40 g/L in agar plates. A 0.5 Check meaning retained diam disk of PDAY fungal growth at 25 ± 2 °C for 2 wk was placed at the center of each plate containing experimental media, using five replicate determinations per culture medium. Plates were then incubated at 25 ± 2 °C for 35 d, and colony radial growth was measured every 7 d, using two cardinal diameters previously drawn on the bottom of each Petri dish, as a reference.

For conidia production, after 35 d incubation, evaluation of radial growth was compared among cultures. For this, 10 mL of distilled water were added on top of each culture to suspend the produced conidia, which were scraped and spread with a bacteriological loop, and one milliliter of residual suspension was taken with a sterile pipette for conidia counting in a Neubauer chamber (Hausser Scientific, Blue Bell, PA, USA).

In a second experiment, only radial growth and sporulation (conidia formation) were evaluated, testing wheat bran and amaranth flour as supplements at 2%, 3%, 4%, and 5%, as explained above.

### 4.2. Conidia Production on Oat

Conidia production as biphasic culture, using the same substrates, protein sources and conditions as described above, were evaluated in two-step solid cultures.

### 4.3. Preparation of Primary Inoculum and Solid Substrates

Conidia inoculum for each strain was prepared by adding 10 mL of sterile water to *Hirsutella citriformis* agar culture, resulting in abundant conidia, which were suspended by light scraping with a bacteriological plastic loop. The suspension was then collected and vigorously mixed for 5 min by vortexing at high speed (level 7), after which the concentration was adjusted to 1 × 10^6^ conidia/mL.

### 4.4. Solid Culture First Step

Oat grains used as solid substrates for spore production were washed three times with water to remove external particles, after which 100 g of substrate were placed in 500 mL plastic containers and soaked in 200 mL of water for 24 h. Next, water was drained, and 4% wheat bran was added and sterilized twice for 30 min in a 24 h period, after which 16 mL of a suspension containing 1 × 10^6^ conidia/g was used as inoculum and containers incubated at 25 ± 2 °C for 21 d.

### 4.5. Solid Culture Second Step

Three days before inoculum preparation, conidia viability and culture purity were determined. Conidia germination was evaluated as previously reported [12]. Cultures made in plastic containers were used to prepare solid culture inoculum in polypropylene bags (40 × 25 cm). For this, conidia were suspended by adding distilled water to containers, vigorously mixed for 5 min by vortexing at high speed (level 7), and adjusted to 1 × 10^6^ conidia/mL.

Polypropylene bags (2 kg) were prepared by adhesion to the top of a 2.5 L plastic bottle and adding 500 g of oat as substrate, supplemented with 2% or 4% wheat brand and/or amaranth (pre-soaked in water for 24 h). The sterilization method was the same used for culturing in plastic containers. Next, 18 mL of a suspension containing 1 × 10^6^ conidia/mL was added as inoculum. Polypropylene bags were then incubated at 25 ± 2 °C for 14 d, after which, 1 g of each fungal strain grown on oat was removed to suspend the produced conidia in 9 mL of water, vortexed at high speed (level 7) for 5 min, and released conidia quantified in a Neubauer chamber. This experiment was performed three times with four replicate determinations, on separate dates.

### 4.6. Hirsutella Citriformis Gum Analysis

To evaluate the crude gum, mucilage produced by each *Hirsutella citriformis* strain in solid culture was extracted with a micropipette. *Hirsutella* OP-Hir-10 strain was isolated from mycosed *Diaphorina citri* adults presenting synnemata. Insect specimens were collected in Uman Xtepen, Yucatan, Mexico. The monoconidial strain was directly obtained from sporulated synnemata. Strains remained active in potato dextrose agar medium containing 1% yeast extract (PDAY; Difco Laboratories, Sparks, MD, USA) and were stored for extended periods in water.

Gum was immediately frozen and stored a −20 °C, until use. Gum solutions were sterilized by autoclaving 15 min at 15 lb and 120 °C, after which they were vortexed for gum homogenization. For protein bands analysis, samples were homogenized in a test-tube homogenizer, with 5 mL of buffer (50 mM Tris-HCl, pH 8.0) per gram of sample, at 4 °C. Samples included (a) supernatant from strain liquid cultures, gum dissolved in isopropanol 1:1, and strain mycelium and (b) gummous material dissolved in isopropanol 1:1 and sonicated (five pulses/40 W/30 s) with or without mycelium, and molecular size markers. Once homogenized, samples were centrifuged for 5 min at 12,000× *g* and 4 °C. The resulting pellet was discarded and supernatant was processed for immediate analysis. We then performed SDS-PAGE to detect protein products bands from *Hirsutella citriformis*. Samples were mixed with loading buffer and electrophoresed at a constant 35 mA at 4 °C, after which gel was stained in 0.1% Coomassie brilliant blue R-250 in 30% (*v*/*v*) methanol and 10% (*v*/*v*) acetic acid, was distained in these solvents (to show protein bands) and interpreted for protein size [34].

### 4.7. Hirsutella Citriformis Conidia Formulation

For *Hirsutella citriformis* gum samples collection, 500 mL flasks containing 250 mL of potato dextrose broth with 1% yeast extract were inoculated with 3 cm^2^ of *H. citriformis* PDAY fungal grown on agar. Flasks were incubated for 9 d at 25 ± 2 °C and 150 rpm. To separate the mycelium from the medium, cultures were centrifuged for 5 min at 10,000 rpm, the supernatant was transferred to a beaker, and two volumes of isopropanol were added. This mixture was kept at rest for 12 h, performing a second isopropanol wash for 12 h. The resulting gum was dried using an infrared moisture balance (Mettler Toledo, Thermo Fisher Scientific, Monterrey, NL, Mexico).

*Hirsutella citriformis* conidia formulation ingredients included conidia (active ingredient), *Acacia* gum (Desarrollo de Especialidades Químicas, SA de CV, Monterrey, NL, Mexico), and *H. citriformis* gum, mixed in distilled water. To formulate conidia, emulsions were prepared using 0.5 g of *Hirsutella* or *Acacia* gums mixed with 7.8 mL of distilled water to reach a final 0.5% *w*/*v* gum in the emulsion. Once cold, conidia were added to the gum solution to reach a final concentration of 1 × 10^7^ conidia/mL.

### 4.8. Laboratory Bioassay

*Hirsutella citriformis* formulated conidia effectiveness to biocontrol *Diaphorina citri* adults was evaluated under laboratory conditions, using 1 × 10^7^ conidia/mL and adding gums as adherent. Bioassays consisted of five replicate determinations against 15 *Diaphorina citri* adults per treatment, applying the treatments absolute control (TA), *Acacia* gum control (TGA), *Hirsutella* gum control (TGH), conidia formulated with *Acacia* gum (HGA), conidia formulated with *Hirsutella* gum (HGH), and distilled water as the negative control. All treatments were applied by direct spraying, using 0.3 mL on 15 *Diaphorina citri* adults placed on citrus leaves. Exposed insects were transferred inside experimental chambers at 27 ± 2 °C and evaluated every three days for up to 21 d, after which insect mortality was recorded.

### 4.9. First Field Assay

Conidia used for field trials was produced on rice. To achieve this, rice was washed three times, soaked in hot water for 30 min, drained thoroughly, and browned with 20 mL of vegetable oil/Kg. Next, 300 mL of water/Kg was added and cooked until evaporation. Sets of 50 g of rice were placed in 500 mL plastic containers and sterilized for 20 min at 121 °C, after which 8 mL of a suspension of OP-Hir-10 *Hirsutella citriformis* 1 × 10^6^ conidia/mL were added to each container. Cultures were then incubated at 25 ± 2 °C for 21 d and conidia were harvested [24]. Conidia viability was confirmed by the colony-forming units (CFU) assay [24].

Field trials were performed on *Citrus sinensis* (L.) Osbeck ‘Valencia’ (Rutaceae) in Montemorelos, NL, Mexico (25°17′12.69″ N, 99°55′39.99″ W). Bioassays started on 1 October and ended on 22 October 2020. Recorded average temperature was 23 °C (18 °C to 28 °C), 3.0 mm precipitation, and 69% relative humidity. *Diaphorina citri* adults were collected with a buccal plastic aspirator and transported to our laboratory facility inside a manual cooler.

Fungi were grown on agar media supplemented with 4% wheat bran and 4% amaranth. Conidia cultured on plates were harvested in 10 mL of sterile distilled water. This suspension was mixed for 5 min, after which 0.5% *w*/*v Acacia* gum (Desarrollo de Especialidades Químicas, S. A. de C. V. Monterrey, NL, Mexico) or 0.5% *w*/*v Hirsutella* gum was added to serve as adherent. The concentration was then adjusted to 1 × 10^7^ conidia/mL. For the untreated control, the solution contained only sterile water and 0.5% *w*/*v Acacia* or *Hirsutella* gums.

Treatments were sprayed to 15 healthy and anesthetized insects per replicate determination, which were placed on absorbent paper in a plastic tray. Spraying of the conidia suspension was performed with a commercial manual sprayer (15 mL volume and 0.3 mm nozzle diameter) at approximately 15 cm distance from the insects. Conidia were sprayed three consecutive times for each array of insects. Inoculated insects were placed in 20 cm × 20 cm bags made with mousseline cloth.

The experiment was completely randomized, including the following treatments: (1) OP-Hir-10 fungal suspension containing 1 × 10^7^ conidia/mL, added with 0.5% *w*/*v Acacia* gum, (2) OP-Hir-10 fungal suspension containing 1 × 10^7^ conidia/mL, added with 0.5% *w*/*v Hirsutella* gum, (3) untreated control sprayed with 0.5% *Acacia* gum solution, (4) untreated control sprayed with 0.5% *Hirsutella* gum and (5) absolute untreated control. Bagged *Diaphorina citri* adults were transferred to flush shoots free of *D. citri* and natural enemies in the morning. Six replicate determinations (considering each shoot as a replicate) were performed for each treatment. Mortality evaluation was performed 21 d after inoculation.

### 4.10. Second Field Assay

Second field trials were also performed on *Citrus sinensis* in Montemorelos, Nuevo Leon. Bioassays started on 6 October and ended on 27 October 2021. The average temperature was 22.8 °C (18 °C to 28 °C), 3.3 mm precipitation and 76% relative humidity. Similarly, *Diaphorina citri* adults were collected and transported to our facility in a manual cooler.

*Hirsutella citriformis* conidia effectiveness, in the same formulations described in the laboratory bioassay, was analyzed under field conditions. Treatments were applied on 15 healthy and anesthetized adults per replicate determination by spraying, using the same dose as for laboratory bioassay. Exposed insects were placed in 20 cm × 20 cm bags made with mousseline cloth, which were placed in tender citrus tree shoots. Mortality evaluation was performed 21 d after inoculation.

### 4.11. Statistical Analysis

Data for mycelial growth and conidial yield were subjected to ANOVA. Means were compared with the Tukey test, using the software IBM SPSS Statistics Version 21 (SPSS, Inc., Chicago, IL, USA). Germination and conidia production data were analyzed by IBM SPSS Statistics Version 21, using a completely randomized design. One-way ANOVA was used to analyze mortality data from treatments of the different bioassays. With data recorded from the laboratory bioassays, the lethal time from treatments was calculated using the Kaplan-Meier test.

## 5. Conclusions

We evaluated diverse substrates as protein sources for *Hirsutella citriformis* conidia production and measured their effectivity after formulating them with *Acacia* and *Hirsutella* gums. The oat and wheat bran combination induced the highest *Hirsutella* conidia production. As a supplement, wheat bran is an inexpensive nutrient source with a 0.71:1 nitrogen: carbohydrate ratio plus minerals and vitamins, which is recommended for *Hirsutella citriformis* biocontrol agent mass production. All tested formulations caused *Diaphorina citri* adult mortality under laboratory and field conditions. In addition, *Hirsutella* gum showed a synergistic effect with *Hirsutella citriformis* for *Diaphorina citri* adults biocontrol on citrus, since the formulation containing conidia and *Hirsutella* gum showed the highest effectiveness to control *D. citri* adults.

## Figures and Tables

**Table 1:** *Hirsutella citriformis* strain INIFAP-Hir-2 radial growth and conidiation ^1^.

	Flours		Extracts	
Protein	Growth (cm) ^2^	Conidiation ^3^ (1 × 10^6^)	Growth (cm) ^2^	Conidiation ^3^ (1 × 10^6^)
2% Soybean	2.9 ± 0.09 ^d^	25.1 ± 1.0 ^b^	2.5 ± 0.02 ^d,e^	13.1 ± 1.1 ^h^
4% Soybean	3.6 ± 0.02 ^a,b^	23.1 ± 1.4 ^b,c^	3.3 ± 0.03 ^b,c^	12.1 ± 1 ^j^
2% Amaranth	3.4 ± 0.13 ^a,b^	24.1 ± 1.1 ^b,c^	3.4 ± 0.16 ^a,b^	13.6 ± 0.7 ^h^
4% Amaranth	3.6 ± 0.02 ^a,b^	19.4 ± 1.8 ^b,c,d^	3.4 ± 0.18 ^a,b^	21.2 ± 0.5 ^b,c,d^
2% Quinoa	3.5 ± 0.06 ^a,b^	21.9 ± 1.4 ^b,c,d^	3.4 ± 0.09 ^a,b^	12.5 ± 0.8 ^j^
4% Quinoa	3.6 ± 0.05 ^a,b^	20.5 ± 0.8 ^b,c,d^	3.6 ± 0.05 ^a,b^	20.6 ± 0.6 ^b,c,d^
2% Wheat germ	3.5 ± 0.1 ^a,b^	12.9 ± 1.8 ^i^	3.4 ± 0.08 ^a,b^	23.9 ± 1.1 ^b,c,d^
4% Wheat germ	3.6 ± 0.03 ^a,b^	15.3 ± 0.5 ^f^	3.7 ± 0.03 ^a,b^	19.6 ± 1.0 ^b,c,d^
2% Wheat bran	3.0 ± 0.1 ^a,b^	24.7 ± 1.2 ^b,c^	3.7 ± 0.04 ^a^	18.2 ± 0.9 ^c,d^
4% Wheat bran	3.4 ± 0.13 ^a,b^	36.9 ± 2.8 ^a^	3.6 ± 0.06 ^f^	18.4 ± 0.4 ^c,d^
2% Pumpkin seed	2.9 ± 0.05 ^c,d^	14.6 ± 0.4 ^g^	3.5 ± 0.02 ^a,b^	16.3 ± 0.8 ^e^
4% Pumpkin seed	3.0 ± 0.01 ^e^	18.4 ± 0.8 ^c,d^	3.6 ± 0.02 ^a,b^	17.1 ± 0.2 ^d,e^
PDAY	3.6 ± 0.02 ^a,b^	17.5 ± 0.7 ^d,e^		

^1^ After 35 d incubation at 25 °C on agar culture medium. ^2^ Different letters (growth) indicate significant differences (Tukey α = 0.05). ^3^ Different letters (conidiation) indicate significant differences (Tukey α = 0.05).

**Table 2:** Protein content of grains and seeds evaluated as supplement for *Hirsutella citriformis* strains growth in solid culture.

Protein Source	Wheat Bran	Wheat Germ	Soybean	Amaranth	Quinoa	Pumpkin Seeds
Protein content (g/100 g)	17.3	31	36	13.56	14.12	24.5

## Data Availability

Not applicable.

## Supplementary Materials

The following supporting information can be downloaded at: https://www.mdpi.com/article/10.3390/plants12061409/s1, Table S1: *Diaphorina citri* adults median lethal time (LT50) (95% confidence limits) (days) after exposed to several formulations; Figure S1: *Diaphorina citri* adults survival percentage average in laboratory bioassay after *Hirsutellla citriformis* formulated conidia application by spraying.
