# Supplementary material for: Improved Diaphorina citri (Hemiptera: Liviidae) Adults Biocontrol in Citrus by Hirsutella citriformis (Hypocreales: Ophiocordycipitaceae) Gum-Enhanced Conidia Formulation
Source: Plants (Basel). 2023 Mar 22;12(6):1409. doi: 10.3390/plants12061409 (PMC10055025; doi:10.3390/plants12061409)
Supplement: Supplementary file 1 [file plants-12-01409-s001.zip › plants-2240101-supplementary.pdf]

Table S1. ***Diaphorina citri ADULTS*** **median lethal time (LT_{50}) (95% confidence limits) (days) after exposed to several formulations** [1]

Formulations				
**TA**	**TGA**	**TGH**	**HGA**	**HGH**
16.528 (15.72-17.38)	17.224 (15.86-18.59)	15.024 (13.56-16.53)	16.391 (15.32-17.47)	13.2 (11.37-15.03)

[1] TA = absolute control, TGA = *Acacia* gum control, TGH = *Hirsutella* gum control, HGA = formulation with *Acacia* gum, HGH = formulation with *Hirsutella* gum. Based on the confidence limits, the significant lowest *Diaphorina citri* lethal time was observed by the HGH formulation exposure (Kaplan-Meier analysis).

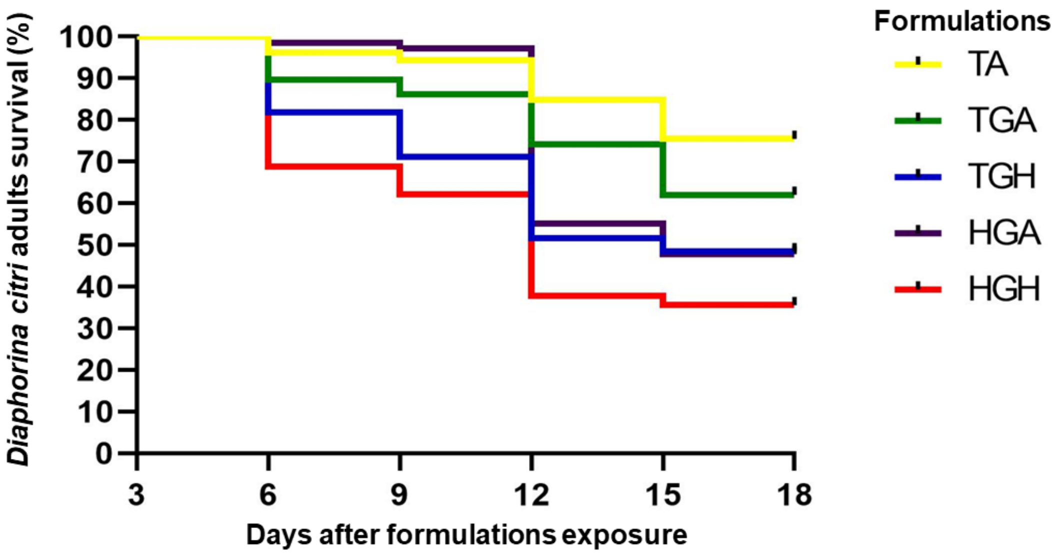

Figure S1. *Diaphorina citri* adults' survival percentage average in laboratory bioassay after *Hirsutella citriformis* formulated conidia application by spraying. TA = absolute control, TGA = *Acacia* gum control, TGH = *Hirsutella* gum control, HGA = formulation with *Acacia* gum, HGH = formulation with *Hirsutella* gumthe Hirsutella gum control (TGH) presented a mortality of 57.8%. (Kaplan-Meier analysis)
